# Motion Synchronous Composite Decoupling with Fewer Sensors on Multichannel Hydraulic Force Control for Aircraft Structural Loading Test System

**DOI:** 10.3390/s18114050

**Published:** 2018-11-20

**Authors:** Yaoxing Shang, Ning Bai, Lingzhi Jiao, Nan Yao, Shuai Wu, Zongxia Jiao

**Affiliations:** National Key laboratory on Aircraft Fight Control, School of Automation Science and Electrical Engineering, Beihang University, Beijing 100191, China; syx@buaa.edu.cn (Y.S.); baining@buaa.edu.cn (N.B.); jiaolingzhi2016@buaa.edu.cn (L.J.); yaonan@buaa.edu.cn (N.Y.); zxjiao@buaa.edu.cn (Z.J.)

**Keywords:** structural test, load senor, hydraulic loading system, fatigue test, force control, aircraft strain gauges, coupling

## Abstract

The aircraft full-scale fatigue test is widely used in the modern aircraft industry for the safety of flight. Generally, the aircraft full-scale fatigue test is achieved by structural loading; multiple hydraulic actuators are used to apply load for force control. The fatigue loading test takes approximately several years. A key challenge is how to accelerate the loading frequency to shorten the total test time. Nevertheless, when pluralities of hydraulic actuator simultaneously increase the loading frequency, the mutual coupling force from the low rigidity of the aircraft structure will cause a large loading error, meaning that the test cannot be implemented. Although it is possible to reduce error by adding sensors, the force sensors need to connect several kilometers of cable. This paper proposed a novel motion synchronous composite decoupling control strategy with fewer sensors. The control method compensates the negative coupling effect of the channels by integrating the command signals and feedback signals of all channels. It can suppress coupling force and reduce errors at higher frequencies, thereby shortening the experiment time. Opposed to traditional decoupling control methods, advantages of this strategy are that it only needs force sensors and it does not need additional displacement or velocity and acceleration sensors to collect state variables for building the state space. Furthermore, it has been experimentally verified that the new motion synchronous composite decoupling control method can indeed guarantee sufficient control accuracy when the test frequency is increased. The method has great economic significance for shortening test duration.

## 1. Introduction

The structure of an aircraft forms the basis of aircraft flight safety. In order to guarantee flight safety and predict the service life of the aircraft structure accurately, structural fatigue loading tests [1] must be carried out on the ground. According to the classic “bottom-top” approach [2,3], the aircraft full-scale fatigue test is located in the top of the “bottom-top” approach, which is the closest to the real fight situation. The FAA (Federal Aviation Administration), EASA (European Aviation Safety Administration), and CAAC (Civil Aviation Administration of China) have all clearly stated in their respective certification regulations that the full-scale fatigue test verification report must be provided.

At present, the aircraft full-scale fatigue test is executed by multichannel hydraulic coordinated loading. The full-aircraft uses dozens to hundreds of hydraulic pressure control systems to apply an alternating load to simulate the huge load of an aircraft flying in the air [4]. Firstly, a hydraulic actuator is arranged at each test point of the aircraft structure which is equipped with a load sensor for force closed-loop control. Secondly, the aircraft is covered with approximately 10,000 strain gauges. These strain gauges monitor whether the structural deformation of the aircraft meets the design requirements.

The current aircraft fatigue loading test urgently needs to shorten the time: It takes 2 to 6 years [5] to complete. Because the average life of modern civil aircraft is as high as 60,000 flight hours multiple life tests are required in the test [6,7]. The key to influencing the test time now is the limitation of the loading frequency and the strong coupling between the channels [8,9].

**Figure 1:**
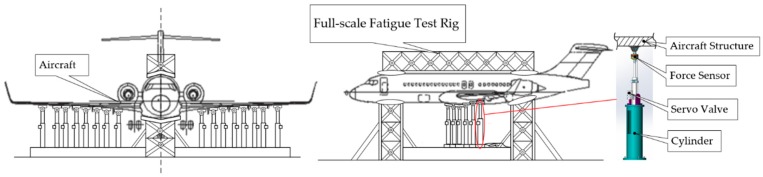
Typical full-scale fatigue test.

Due to the low rigidity of the aircraft structure and the large deformation, the current loading frequency is only 0.1 Hz. Using a typical aircraft airfoil structural strength test as an example, the span of the airfoil tip and the airfoil root is as long as several tens of meters, and the airfoil tip is deformed by several meters [10]. Typical full-scale fatigue test force loading figure as shown in Figure 1. Accelerating the load frequency slightly will exacerbate the load–force coupling between the different loading channels, causing the loading error of the loading force control to be out of tolerance [11].

Therefore, how to overcome the coupling effect between the loading channels and achieve accurate and unified loading of multiple channels has become a major problem in this field. The key to overcome this problem is how to only use force sensors for the decoupling control [12,13] of dozens of channels. Because the size of the civil aircraft is very large, only the cable of the force sensors is several kilometers in the fatigue test, and the addition of a new sensor is hardly allowed. Many decoupling methods exist today, such as state space decoupling [14], which requires the acquisition of process variables such as position, velocity, and even acceleration of each loading point in addition to pressure sampling. A large number of different types of sensors are not only unbearable for cable scale, but also exacerbate electromagnetic compatibility (EMC) problems, and the huge data computing load will limit the real-time of the control system.

Therefore, this paper proposes a novel synchronous composite decoupling control method with less sensors based on multichannel servo control signals. The innovation of this method is to combine the control signals of all other channels with the given signal of the current channel, and use this compound signal as the feed forward input signal of the current channel to compensate the servo output of each single channel to eliminate the coupling force interference between channels.

This method need not the complex coupling link between the simulated load force and the deformation displacement, omitting the use of state variables such as position, velocity, and acceleration, and avoiding the signal noise caused by the numerical differentiation of the position signal. The feed forward compensation signal used in this method has obvious advanced predictability, which can ensure the fastness and consistency of multichannel loading of digital discrete control.

## 2. Analysis of the Loading Coupling

### 2.1. Principle of Displacement Overlapping

In the actual aircraft full-scale fatigue test, the loading method of various parts is also varied. However, the most common high-aspect-ratio airfoil is simple in shape. The high-aspect-ratio airfoil loading can be approximated as a one-dimensional linear loading, so the loading points are arranged in a straight line in space. By analyzing the relationship between the airfoil and the fuselage, and the displacement of the airfoil root during loading is almost negligible. Therefore, the airfoil can be regarded as a cantilever beam.

**Figure 2:**
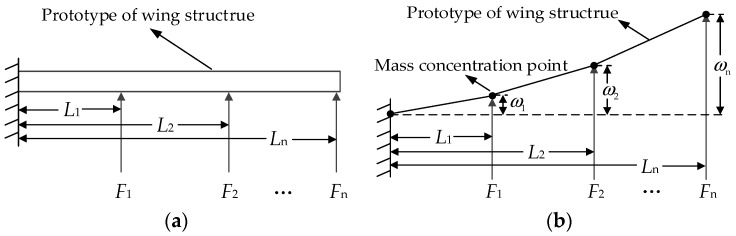
Loading model of the structural test. (**a**) shows a simplified model of the airfoil. (**b**) shows the deflection of the model at each loading point.

Mathematical modeling of the multi-point force of the cantilever beam is carried out next. Without losing the generality, the entire cantilever beam is divided into three parts which are loaded through three channels; assuming that the loading channel produces a vertical upward force. The cantilever beam is also bent in the vertical direction at the loading point; there is no bending or twisting in other directions. According to the material mechanics, the cantilever beam has a small deformation within a certain range, and the stress subjected to the stress does not exceed the maximum stress. Therefore, the deflection curve of the cantilever beam can be approximated by a linear differential equation. When three loads are simultaneously loaded on the cantilever beam, the deflection curve of the cantilever beam can be regarded as a linear superposition combination of different deflection curve equations generated by a single load acting on different loading points of the cantilever beam according to this relationship. When the linearly distributed load force acts on the cantilever beam [15], the deflection curve equation of the cantilever beam can be obtained by the superposition principle [16] of the deflections. The model is shown in Figure 2.

In Figure 2a, the loading object is the cantilever beam which is a simplified model representing the airfoil. The vertical upward arrows represent that the hydraulic cylinders apply force to different position of cantilever beam at the same time. The cantilever beam is naturally divided into n pieces according to the actual position of the loading point. The mass of each section is the percentage of the length of the section multiplied by the total mass. $F_{i}$ is the loading force of loading channel *i*. $L_{i}$ represents the distance from loading point *i* to the fixed side of the cantilever beam. In Figure 2b, $\omega_{i}$ represents the deflection of the loading point *i*. Here we assume *n* = 3 and *i* = 1, 2, 3.

According to the material mechanics, when only the load force $F_{1}$ acts on the loading point 1, the deflection of point 1 is

(1) $$\;\omega_{11}=\frac{F_{1}L_{1}^{3}}{3EI},\;$$

where *EI* is the bending stiffness of the cantilever beam and the deflection of point 2 is

(2) $$\;\omega_{12}=\frac{F_{1}L_{1}^{3}}{3EI}\left(3L_{2}-L_{1}\right),\;$$

The deflection produced by $F_{1}$ at the point 2 is

(3) $$\;\omega_{13}=\frac{F_{1}L_{1}^{3}}{3EI}\left(3L_{3}-L_{1}\right),\;$$

The deflections generated by$\;F_{1}$, $F_{2}$, and $F_{3}$ at the action points 1, 2, and 3 can be calculated separately.

According to the relevant principles of material mechanics, when only $F_{i}$ is applied on the cantilever beam, the deflection [17] of the loading point *j* is given as

(4) $$\;\omega_{ij}=F_{i}\delta_{ij},\;$$

where $\delta_{ij}$ is the relative flexibility from channel *i* to channel *j*.

According to the superposition principle, when total *n* points are loading at the same time, $\omega_{j}$ indicating the deflection of the loading point *j* is given as

(5) $$\;\omega_{j}=\sum_{i}^{n}\omega_{ij},\;$$

Thus, the deflection vector ***ω*** can be given as

(6) $$\;\omega =\left|\delta_{ij}\right|F,\;$$

where $\left|\;\delta_{ij}\right|$ (*i* = 1, 2, …, *j* = 1, 2, …, *n*) is the flexibility matrix of the cantilever beam. ***F*** represents the vector of loading force.

If *i* and *j* are all equal to 3:

(7) $$\left[\begin{array}{c} \omega_{1}\\ \omega_{2}\\ \omega_{3} \end{array}\right]=\;\left[\begin{array}{ccc} \frac{L_{1}^{3}}{3EI} & \frac{L_{1}^{2}\left(3L_{2}-L_{1}\right)}{6EI} & \frac{L_{1}^{2}\left(3L_{3}-L_{1}\right)}{6EI}\\ \frac{L_{1}^{2}\left(3L_{2}-L_{1}\right)}{6EI} & \frac{L_{2}^{3}}{3EI} & \frac{L_{2}^{2}\left(3L_{3}-L_{2}\right)}{6EI}\\ \frac{L_{1}^{2}\left(3L_{3}-L_{1}\right)}{6EI} & \frac{L_{2}^{2}\left(3L_{3}-L_{2}\right)}{6EI} & \frac{L_{3}^{3}}{3EI} \end{array}\right]\;\left[\begin{array}{c} F_{1}\\ F_{2}\\ F_{3} \end{array}\right].\;$$

The above equation reveals the basic coupling relationship of the cantilever beam linear distributed low frequency and small amplitude loading.

### 2.2. Mass Concentration Method

The distributed mass of the cantilever beam can be transformed into focus through mass concentration method [18]. Thus, the infinite freedom degree of the system can be transformed into multi-degree of freedom and the calculation can be simplified. According to the static equivalent principle, the concentrated gravity is equivalent to the origin.

The mass strip with uniform distribution is divided into n parts in accordance with the practical loading points. The mass of part *i* is *m_i_*. In light of the static equivalent principle, the distributed mass of a part can be transformed into the concentrated mass of both ends. The concentrated mass of an end is *m_i_*/2. Two contiguous concentrated mass points constitute one concentrated mass in loading point, whose mass is (*m_i_* + *m_i_*_+ 1_)/2. After the transformation of concentrated mass, the loading model of the system with deformation is represented in Figure 2b.

Here we assume *n* = 3, and the masses of loading points 1, 2, and 3 are 1/3, 1/3, and 1/6 of the total mass, respectively.

### 2.3. Analysis of the Loading Coupling

When multiple channels are co-loaded, loading channels *i* and *j* are selected. While channel *i* is loaded, the channel *j* is also loaded. The coupling effect of the force necessarily causes the point *i* to produce additional deflection. This extra deflection is multiplied by the stiffness of the point *i* itself, which creates an additional force from the channel *j* in the channel *i*. It can be simply understood that the force of the channel *j* will be converted to point *i* by a certain proportional coefficient. This force is linearly added to the loading force of channel *i* to produce a displacement at the loading point *i*.

The proportional parameter can be expressed as a linear coupling parameter of the cantilever beam, which is called a structural parameter. Specifically, it can be expressed as the ratio of *j* channel to *i* channel relative flexibility and *i* channel absolute compliance. The structure parameter $K_{ij}$ can be given as

(8) $$\;\left|K_{ij}=\delta_{i}/\delta_{ij}\right|,\;$$

where $\delta_{i}$ is the absolute compliance, and $\delta_{ij}$ is the relative flexibility of each loading point.

Obviously, the force of the channel *j* coupling to the point *i* can be expressed as the structural parameters $K_{ij}$ multiplied by the loading force of channel *j*.

To express more clearly, if *i* is equal to 1, 2, and 3, respectively, the absolute flexibility matrix of the cantilever beam is obtained as follows

(9) $$\left[\begin{array}{ccc} \frac{L_{1}^{3}}{3EI} & \frac{L_{1}^{3}}{3EI} & \frac{L_{1}^{3}}{3EI}\\ \frac{L_{2}^{3}}{3EI} & \frac{L_{2}^{3}}{3EI} & \frac{L_{2}^{3}}{3EI}\\ \frac{L_{3}^{3}}{3EI} & \frac{L_{3}^{3}}{3EI} & \frac{L_{3}^{3}}{3EI} \end{array}\right],$$

Combining the $\delta_{ij}$ generated by (6), the dimensionless matrix with respect to the structural parameter $K_{ij}$ is (*i*, *j* = 1, 2, 3)

(10) $$\left[\begin{array}{ccc} 1 & \frac{3L_{2}-L_{1}}{2L_{1}} & \frac{3L_{3}-L_{1}}{2L_{1}}\\ \frac{L_{1}^{2}\left(3L_{2}-L_{1}\right)}{2L_{2}^{3}} & 1 & \frac{3L_{3}-L_{2}}{2L_{2}}\\ \frac{L_{1}^{2}\left(3L_{3}-L_{1}\right)}{2L_{3}^{3}} & \frac{L_{2}^{2}\left(3L_{3}-L_{2}\right)}{2L_{3}^{3}} & 1 \end{array}\right],$$

Through the above analysis, the matrix can be approximated as a coefficient matrix in which the loading forces between the multiple channels.

Establishing the coupling coefficient matrix has revealed the relationship between the loading points of the cantilever beam when the multichannel load force is simultaneously loaded. However, in order to further simulate the true connection relationship between the servo actuator and the loading point in the full-scale fatigue test, it is necessary to establish a dynamic model of the coupling force relationship between the loading points.

It should be noted that it is different from the general static test. Actuator needs to provide bidirectional load for the loading point in the fatigue test of the airfoil structure. So, the actuator must be directly connected to the cantilever beam loading point. Since the amount of loading force must be measured, a force sensor is required between the actuator and the loading point.

Then, the force coupling equation between the force sensor, the hydraulic actuator and the loading point is established.

The dynamic equation can be constructed by combining the coupling forces of other channels and the output force of the own channel actuators as follows

(11) $$\;F_{i}+F_{o}=M_{i}{\ddot{\omega }}_{i}+B_{i}{\dot{\omega }}_{i}+K_{i},\;$$

where $F_{i}$ is the force of channel *i*, $F_{o}$ is the force of other channel, $M_{i}$ is the mass of the concentrated point, $B_{i}$ is the damping coefficient, and $K_{i}$ is the stiffness coefficient. Subscript *i* indicates channel *i* [19,20,21].

Based on the above dynamic equation of single channel and deflection vector ω, the coupled equations of the loading system can be given as

(12) $$\left\{ \begin{array}{l} K_{11}F_{1}+K_{12}F_{2}+\ldots +K_{1n}F_{n}=M_{1}{\ddot{\omega }}_{1}+B_{1}{\dot{\omega }}_{1}+K_{1}\omega_{1}\\ K_{21}F_{1}+K_{22}F_{2}+\ldots +K_{2n}F_{n}=M_{2}{\ddot{\omega }}_{2}+B_{2}{\dot{\omega }}_{2}+K_{2}\omega_{2}\\ \ldots \ldots \\ K_{n1}F_{1}+K_{n2}F_{2}+\ldots +K_{nn}F_{n}=M_{n}{\ddot{\omega }}_{n}+B_{n}{\dot{\omega }}_{n}+K_{n}\omega_{n} \end{array}\right..$$

The above equations of motion reveal the coupling relationship between the actuator and loading point.

The coupling force dynamics equations of the linear multi-loading point coupling coefficient are constructed by exploring the force coupling relationship between different loading points and the dynamic relationship between the actuator and the loading point. It establishes the mathematical model of the control object in the control system. It is the basis for building a mathematical model of the entire control system.

## 3. Modeling of the Loading System

### 3.1. Single Channel Loading System

After the modeling of the control object is completed, the modeling of the system execution object was performed to facilitate the formation of the entire control system. In the full-size fatigue loading test, it will be limited by space. Therefore, the actuator is required to have small volume, large output force, and fast response speed. So, hydraulic valve-controlled cylinders are used as actuating actuators.

**Figure 3:**
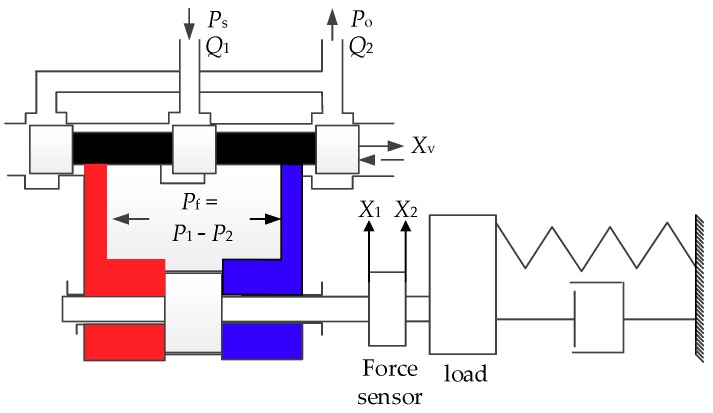
Schematic diagram of the valve-controlled cylinder.

The schematic diagram of the valve-controlled cylinder [22] is shown in Figure 3.

For a control system, the actuator acts as one of several key subsystems. The actuator has many characteristics, which directly determines the performance of the entire system. The mathematical model of the single-channel hydraulic actuator of the motion decoupling control system is constructed [23].

The linear flow equation of actuator system valve is described by

(13) $$\;Q_{L}=K_{q}x_{v}-K_{c}p_{f},\;$$

The Hydraulic cylinder flow continuity equation is subjected to

(14) $$\;Q_{L}=A\frac{dx_{d}}{dt}+\frac{V}{4E}\frac{dp_{f}}{dt}+C_{s}p_{f},\;$$

The equation of forces is expressed by

(15) $$\;p_{f}A=M_{a}\frac{d^{2}x_{d}}{dt^{2}}+B_{a}\frac{dx_{d}}{dt}+K_{a}\left(x_{d}-x_{d}^{\prime }\right),\;$$

According to the analysis of Section 2.3, the piston rod of the hydraulic cylinder is not rigidly connected with the loading point. It is connected by a force sensor with a certain flexibility. In order to ensure that the modeling of the loading system is more accurate, the load is taken as the research object. The load equation of the channel is given as

(16) $$\;K_{a}\left(x_{d}-x_{d}^{\prime }\right)=M_{L}\frac{d^{2}x_{d}^{\prime }}{dt^{2}}+B_{L}\frac{dx_{d}^{\prime }}{dt}+K_{L}x_{d}^{\prime }.\;$$

On the basis of the hydraulic servo valve flow equation, the flow continuity equation of the hydraulic cylinder, the force balance equation of the hydraulic cylinder piston rod, and the loading equation of the loading point, it is easy to construct a single-channel closed-loop hydraulic servo control system with the PID (Proportion-Integral-Derivative) controller [24,25].

### 3.2. Multichannel Loading System

In order to further improve the mathematical modeling of the multichannel force coupled control system, a mathematical model of the force-coupled equations is added based on the multi-servo control channel.

**Figure 4:**
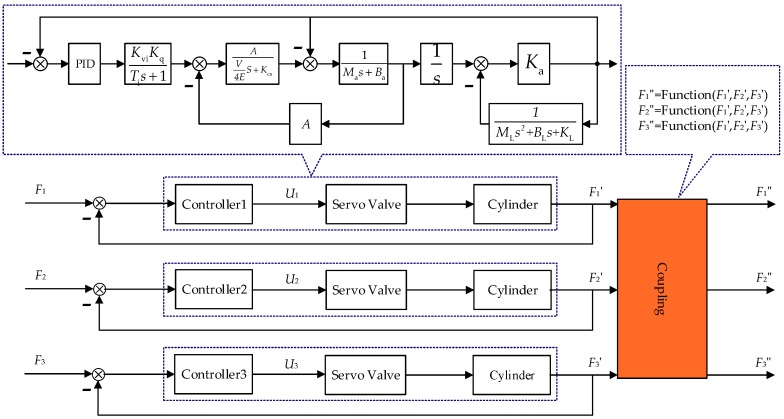
The coupled model of 3-channel loading.

In the multichannel structure loading system [26] the output force of each channel contains the coupling force transmitted by other channels. Based on this, a distributed 3-channel coupled loading model is established and shown in Figure 4.

The three-channel coupling force control system is based on three single-channel valve-controlled cylinder systems. The input signal of the couple model $F^{\prime }$ is the force output signal of each single loading channel. The output of the couple model $F^{\prime \prime }$ can be connected to the load equation and feedback channel of each individual channel. The couple model is the multichannel coupling force dynamics equations deduced from Section 2.3. The above system is the coupling force loading of multichannel. A summary table of all symbols and acronyms is given in Table 1 in the next section.

### 3.3. Analysis of Loading Performance

The MATLAB (MathWorks, R2016b) simulation can be performed on the mathematical model of the multichannel force-coupled servo control system constructed in Section 3.2. It can verify the coupling force interference phenomenon in the multichannel loading process of the airfoil during the aircraft full-scale fatigue test.

The symbol definitions and main parameters of the simulation model are given in Table 1.

**Figure 5:**
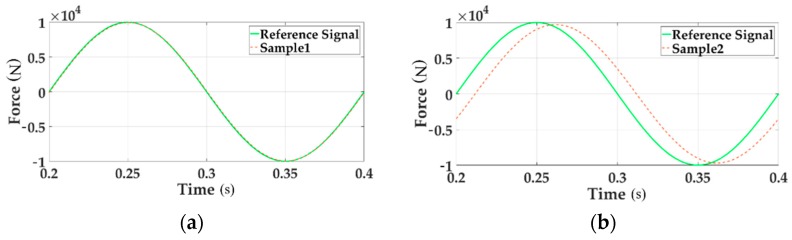
The tracking performances of time domain.

To verify the influence of the coupling disturbance, the contrast tests when single channel loading and multichannel loading are conducted. The dynamic characteristics are shown in Figure 5.

In the above two charts the reference signal represents the command input. The sample signal stands for the system output signal. Figure 5a gives the sinusoidal tracking performance of single loading Channel 1 in the time domain. Figure 5b illustrates the system characteristics of Channel 1 in the case of sinusoidal multichannel loading. By comparing these two figures the tracking accuracy of multi-loading system is seriously reduced. 

**Figure 6:**
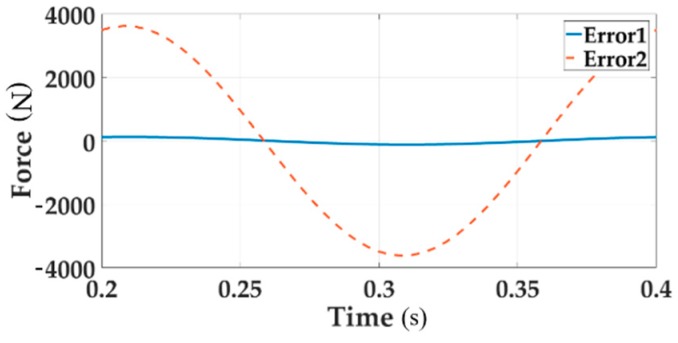
The system error of loading Channel 1 for both single loading and multi-loading.

Figure 6 describes the system error of Channel 1 with single loading and multi-loading, respectively, distinguished with solid line and dotted line.

Figure 6 clearly shows that the tracking performance of the multi-loading system deteriorates when considering a single loading situation. 

**Figure 7:**
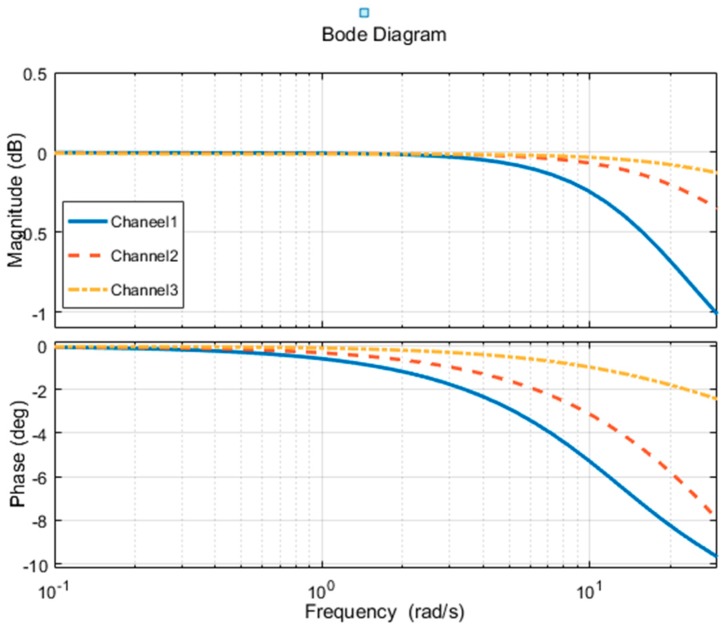
Bode diagram of the coupled force loading system.

The frequency analysis of the coupled force loading system is shown in Figure 7.

In the diagram, the loading point of Channel 1 which is indicated by solid line is close to the “airfoil tip”, the loading point of Channel 2 which is indicated by a dash line is in the middle of the “airfoil” and the loading point of Channel 3 which is indicated by dash-dot line is close to the “airfoil root”. 

When loaded separately, the loading of Channel 3, Channel 2, and Channel 1 is gradually reduced due to the coupling force, which is consistent with the previous stiffness analysis results. That is the closer to the airfoil tip, the lower the stiffness. The phase is more likely to produce hysteresis during loading, and the amplitude is more likely to cause attenuation; for single loading channel, the loading performance also decreases as the loading frequency becomes higher.

By performing time domain and frequency analysis on the coupled system through simulation, the following conclusions can be obtained. The coupling force between the channels will affect the tracking accuracy of the force loading system. The frequency of the loading system increases and the coupling effect between the loading points is intensified. In linear loading, the lower the stiffness is, the stronger the coupling effect becomes. It requires a stronger control algorithm. Thus in order to improve the dynamic characteristics of multi-loading system and reduce the tracking error, it is necessary to design a reasonable decoupling control method.

## 4. The Motion Synchronous Composite Control Method

### 4.1. The Principle of Motion Synchronous Composite Control Method

The foundation of the motion synchronous composite decoupling control method is that when excessive force exists between the active load and the passive load [27] it will be eliminated when the two objects are consistent. When choosing a channel as passive loading, the other active channels offer motion signals to passive loading channel to guarantee the motion of passive loading going along with the others.

In the multi-loading system, which is a typical force control system [28,29], the excessive force is caused by desynchronization among loading channels. This decoupling method is designed from the term of ensuring consistent motion among channels. The motion of every loading channel is controlled by the pressure difference provided from the servo signal. Because the servo signals and force signals have advanced predictive effect, the motion synchronous composite decouple control method fetches these two signals as the current channel’s compensation variable [30]. In this method, some mathematical operations were undertaken to deal with the control signals and the force command signals in order to realize advanced prediction.

From Figure 4 and the correlation equations determined in the first two sections, the output force of a given channel is deduced:

(17) $$K_{a}\left[\left(u\frac{K_{vi}}{1+Ts}K_{q}\frac{A}{\frac{V}{4E}s+K_{cs}}-F\right)\frac{\frac{V}{4E}s+K_{cs}}{\left(M_{a}s+B_{a}\right)\left(\frac{V}{4E}s+K_{cs}\right)+A^{2}}\frac{1}{s}-\frac{F+\sum K_{ij}F_{i}}{M_{l}s^{2}+B_{l}s+K_{l}}\right]=F,$$

where *F* is hydraulic cylinder output force, *u* is the control signal, $\sum K_{ij}F_{i}\;$ represents the coupling force derived from the other channels, and $K_{cs}$ is the sum of $K_{c}$ and $C_{s}$. Reorganize above equation and the expression of the output force is solved as follows

(18) $$F=K_{a}\frac{\frac{K_{vi}K_{q}A\left(M_{l}s^{2}+B_{l}s+K_{l}\right)}{1+Ts}u-\left[\frac{VM_{a}}{4E}s^{2}+\left(K_{cs}M_{a}+\frac{VB_{a}}{4E}\right)s+K_{cs}B_{a}+A^{2}\right]s\sum K_{ij}F_{i}}{\left(M_{l}s^{2}+B_{l}s+K_{l}+K_{a}\right)\left[\left(M_{a}s^{2}+B_{a}s+K_{a}\right)\left(\frac{V}{4E}s+K_{cs}\right)+A^{2}s\right]-\left(\frac{V}{4E}s+K_{cs}\right)K_{a}^{2}},\;$$

then,

(19) $$F=\frac{G_{1}u-G_{2}s\sum K_{ij}F_{i}}{G_{3}},$$

where $G_{1}$, $G_{2}$ and $G_{3}$ are polynomials related to Laplacian operator *s*. It is apparent that the coupling force has negative effect on the output of current loading channel. In the light of theory, it is reliable to eliminate the excessive force by putting a compensation variable $u_{c}$ into the control signal according to following formula.

(20) $$\frac{G_{1}\left(u_{o}+u_{c}\right)-G_{2}s\sum K_{ij}F_{i}}{G_{3}}=\frac{G_{1}u_{o}}{G_{3}},$$

where $u_{o}$ is original control signal and $\;u_{c}$ is the compensation control signal. To make the above formula, we can get

(21) $$\;u_{c}=\frac{G_{2}}{G_{1}}s\sum K_{ij}F_{i},\;$$

It can be known from calculation that the coefficients of many high-order terms about *s* are relatively small compared to the constant terms. So, we directly ignore some of the high-order items, the compensation link can be directly reduced to

(22) $$\;u_{c}=G_{n}\sum K_{ij}F_{i},\;$$

where $G_{n}$ is equal to $G_{2}/G_{1}$. The equation of the control signal with respect to the force output signal is compensated by the above single channel, which is extended to *n* channels. A transfer matrix function of the compensation control signal vector $u_{c}$ with respect to the output force vector ***F*** can be obtained.

(23) $$\;u_{c}=GF,F=G^{-1}u_{c},\;$$

where ***G*** is the transfer matrix of the Laplace. 

The expanded form of the above equation is given as

(24) $$\left\{ \begin{array}{l} u_{c1}=(a_{11}+a_{12}s+\ldots )F_{1}+(b_{11}+b_{12}s+\ldots )F_{2}+\ldots +(k_{11}+k_{12}s+\ldots )F_{n}\\ u_{c2}=(a_{21}+a_{22}s+\ldots )F_{1}+(b_{21}+b_{22}s+\ldots )F_{2}+\ldots +(k_{21}+k_{22}s+\ldots )F_{n}\\ \ldots \ldots \\ u_{cn}=(a_{n1}+a_{n2}s+\ldots )F_{1}+(b_{n1}+b_{n2}s+\ldots )F_{2}+\ldots +(k_{n1}+k_{n2}s+\ldots )F_{n} \end{array}\right.,$$

where $u_{ci}$ is the control signal of channel *i*, $\;F_{i}$ is the output force of channel *i*, and the preceding coefficient is a polynomial about *s*. (*i* = 1, 2, 3, …, *n*) 

In the motion synchronous composite control method, the square term and higher terms of s are neglected. Thus, the following function can be given as

(25) $$\left\{ \begin{array}{l} a_{12}sF_{1}+b_{12}sF_{2}+\ldots +k_{12}sF_{n}=u_{c1}-a_{11}F_{1}-b_{11}F_{2}-\ldots -k_{11}F_{n}\\ a_{22}sF_{1}+b_{22}sF_{2}+\ldots +k_{22}sF_{n}=u_{c2}-a_{21}F_{1}-b_{21}F_{2}-\ldots -k_{21}F_{n}\\ \ldots \ldots \\ a_{n2}sF_{1}+b_{n2}sF_{2}+\ldots +k_{32}sF_{n}=u_{cn}-a_{n1}F_{1}-b_{n1}F_{2}-\ldots -k_{n1}F_{n} \end{array}\right.,$$

where *sF_i_* is took as the new variables of the equation set. The above equations can be solved and $sF_{i}\;$can be expressed as the linear combination of $F_{i}$ and $u_{ci}$. Then once again referring to the previous formula for $u_{c}$:

(26) $$\;u_{c}=\frac{G_{2}}{G_{1}}s\sum K_{ij}F_{i}=\frac{G_{2}}{G_{1}}\sum K_{ij}sF_{i},\;$$

After $sF_{i}$ is plugged into the function of compensation control signal, the compensation control signal $u_{c}$ can be given as

(27) $$\;u_{c}=\frac{G_{2}}{G_{1}}s\sum K_{ij}F_{i}=\frac{G_{2}}{G_{1}}C\left[u_{c},F\right].\;$$

where ***C*** is a constant vector about $K_{ij}$. This realizes the derivation of the decoupling control compensation signal by linear combination of the channel control signal and the output force signal.

**Figure 8:**
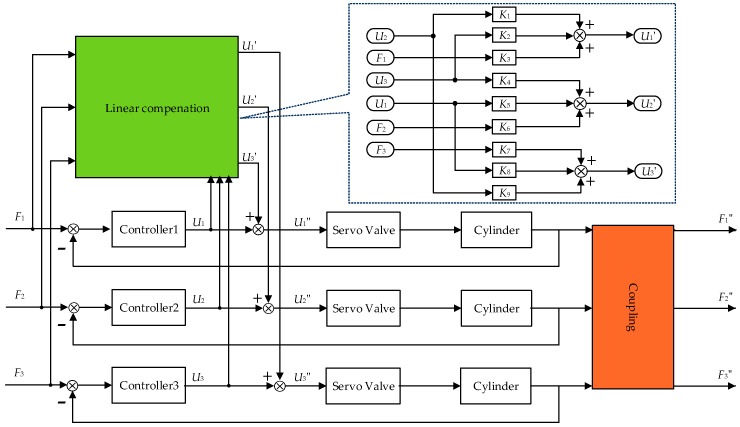
Schematic diagram of motion synchronous composite decoupling control method.

From the above function, the decoupling control compensation signal can be expressed as the linear combination [31] of control signals and force command signals. The multi-loading system with motion synchronous composite decoupling unit is shown in Figure 8.

Due to the control signals and the command signals of each loading channel are selected as the input of the decoupling module, the decoupling signal retains higher order differential information, which is beneficial to improve the decoupling control precision. Moreover, the control signals and command signals have predictive effect and this decoupling method avoids differential processing which is easy to implement on engineering.

### 4.2. The Simulation of Motion Synchronous Composite Control Method

**Figure 9:**
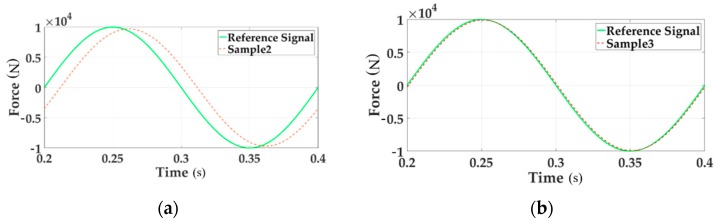
The tracking performances of Channel 1.

In order to verify the compensation controller proposed in this paper, the corresponding simulation verification is given in Figure 9. The reference signal is the force command input of channel 1. The sample signal shows the system output of Channel 1.

**Figure 10:**
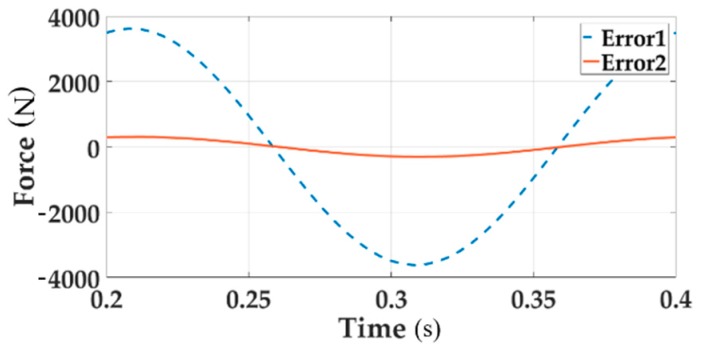
The system error of Channel 1 when multi-loading with and without decoupling module.

Figure 9a gives the sinusoidal tracking performance of Channel 1 when multi-loading without decoupling module in the time domain. Figure 9b gives the sinusoidal tracking performance of Channel 1 when multi-loading with decoupling module in the time domain. Compared above two pictures, it is apparent that the system error after adding the decoupling module shrinks. Thus a conclusion could be obtained that the motion synchronous composite decoupling control method decreases the system error exiting in multi-loading system and improve the dynamic characteristics. The system error is shown in Figure 10.

In Figure 10, the dotted line and the solid line respectively indicates the system error of channel 1 when multi-loading without and with decoupling. From the above time domain analysis, it can be proved that the decoupling control algorithm plays an important role in the multichannel coupled loading system. Motion synchronous composite decoupling method can effectively improve the tracking accuracy of the force and reduce the error.

**Figure 11:**
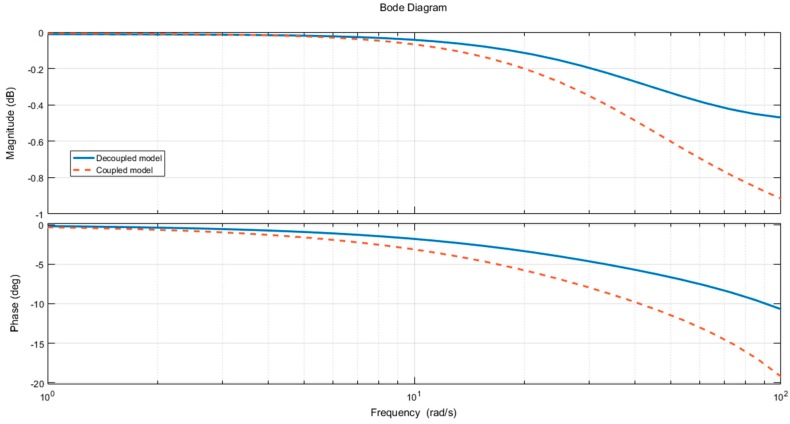
The contrast Bode diagram of decoupling and coupling loading system.

We perform frequency analysis on the decoupling control method. The system’s Bode diagram for decoupling and without decoupling in the control system is shown in Figure 11.

The diagram above is a comparison of the frequency response of the Channel 2. The figure consists of two parts, which represent the amplitude-frequency characteristics and the phase-frequency characteristics. In the simulation of the coupling force system, the frequency response is indicated by the solid line. After the system is added to the decoupling control algorithm, the frequency response curve of the Channel 2 is indicated by dot line. With the increase of frequency, the control characteristics of the decoupling control algorithm have achieved obvious effects in both the amplitude-frequency characteristic and the phase-frequency characteristic.

## 5. Experiment

In the full-scale fatigue test, the test agency usually perform multi-point linear loading on the airfoil. Therefore, the test bench is designed in the same way. According to the determined loading method, the test bench consists of a simplified design of the loading cantilever beam, a supporting rigid frame, a valve-controlled hydraulic cylinder, and a centralized hydraulic source.

**Figure 12:**
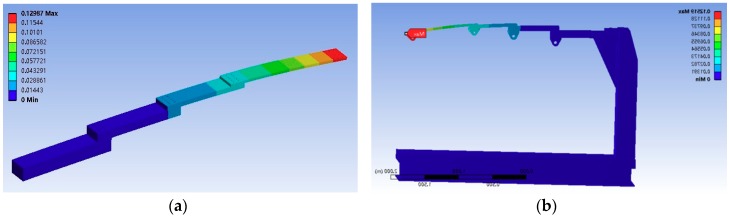
ANSYS simulation for cantilever beam and test bench. (**a**) shows ANSYS simulation for cantilever beam. (**b**) shows ANSYS simulation for test bench.

In order to make the test bench meeting the extensive test requirements and economic considerations of experimental materials, a segmented cantilever beam structure is designed; the material is processed by 40 Cr. We used ANSYS (ANSYS, 18.2) to simulate the loading of the test bench. ASNSYS simulation for cantilever beam and test bench is shown in Figure 12a,b.

In order to simulate the force of the real airfoil, we applied four forces—20 kN, 10 kN, 8 kN, and 3 kN—to the four action points, respectively; the shape variables were 7.2 mm, 14.4 mm, 43 mm, and 129 mm.

To prove that the designed support frame conforms to the constraint form of the separate analysis of the loaded parts, we designed a segmented cantilever beam and frame to be integrated through the earring structure connection. Through ANSYS analysis, we applied 20 kN, 10 kN, 8 kN, and 3 kN loading force again at the corresponding position. The deformation amounts are approximately the same as that of the test piece: 6.8 mm, 13.9 mm, 42 mm, and 125.1 mm.

Then, the modal analysis was carried out on the test bench. Through the results, it was found that the first mode of the test bench was 9.167 Hz. Test frequency is much lower than 9.7 Hz, which excludes the danger of resonance. The test bench designed in this way is a good simulation of the load condition of the aircraft airfoil and meets the purpose of requirements.

**Figure 13:**
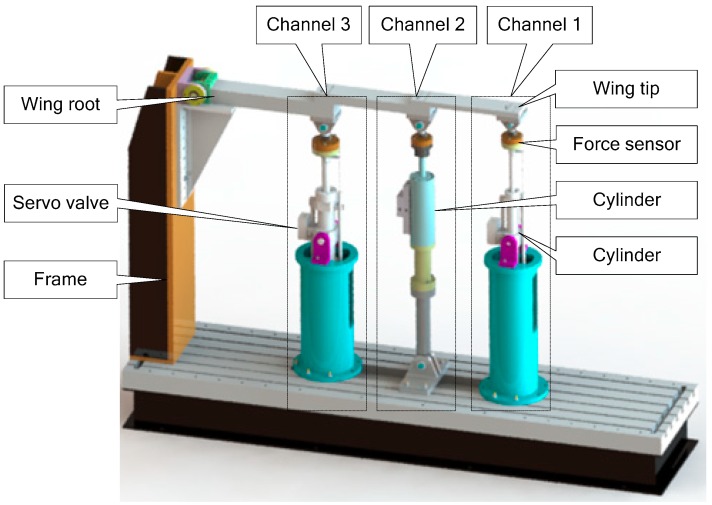
Distributed structure loading test bench.

The hardware of experiment contains valve control cylinders, force tensors, etc., illustrated in Figure 13.

Figure 13 shows the 3 channels loading system. From the tip of the airfoil to the root of the airfoil, the three channels are Channel 1, Channel 2, and Channel 3, respectively. The maximum bearing capacities [32] of the three channels are correspondingly 8000 N, 10,000 N, and 20,000 N.

**Figure 14:**
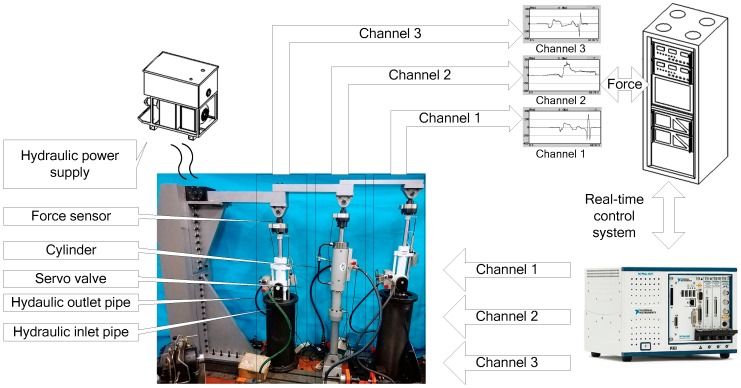
The complete system diagram of the multichannel loading system.

The signal flow of multi-loading system is expressed in Figure 14. 

The software is mainly responsible for collecting relevant electrical signals and bus signals, and transmitting the signals collected in real time to the control system of host computer through TCP; at the same time, the host computer receives the instructions for multichannel and multi-modes switching signals, bus signals, and hardware analog outputs. In this experiment, it is feasible to determine the optimal value of each compensation parameter by experimental trial.

**Figure 15:**
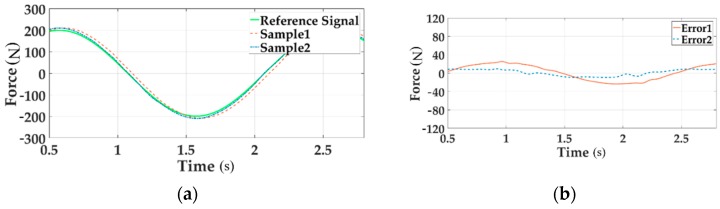
The force tracking experiment of Channel 1 in the time domain.

**Figure 16:**
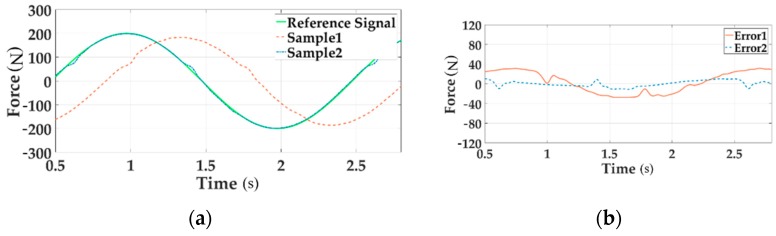
The force tracking experiment of Channel 2 in the time domain.

**Figure 17:**
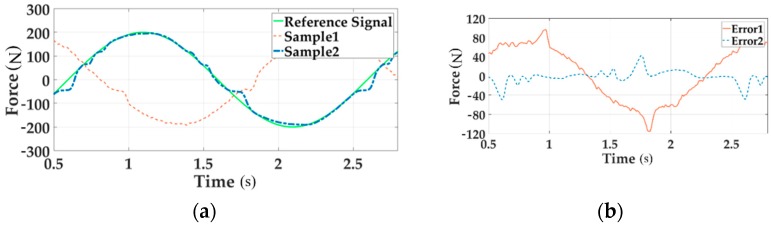
The force tracking experiment of Channel 3 in the time domain.

The real force tracking of all channels under 200 Nm loading at 0.5 Hz before and after adding the decoupling module are shown in Figure 15, Figure 16 and Figure 17.

In these Figures a, the reference signal is the command input, Sample 1 is the output force without decoupling when all three channels are loading, Sample 2 means the output signal with decoupling in the situation of multi-loading. In these Figures b, Error 1 implies the system error without decoupling and Error 2 senses the error with decoupling when all channels load.

As can be seen from the above figures, the motion synchronization composition decoupling method has improved amplitude and phase characteristics of distribute loading system.

## 6. Discussion

This paper solved the coupling force problem in the aircraft full-scale fatigue test. This decoupling control algorithm is especially suitable for multichannel loading of the airfoil. The idea of this paper is derived from the use of the steering angular velocity command in the load simulator system to obtain the synchronization signal, so as to eliminate the redundant force. 

In the load simulator system, the loading system is subject to the active motion interference of the loaded object (such as the steering gear) during the tracking load spectrum process. The first problem in improving the loading accuracy is to eliminate the motion disturbance from the loaded object, which belongs to the category of synchronous control question. The speed synchronization control method introduces valve control signals for speed compensation, avoiding dependence on the displacement sensor signal. This method could improve the ability to eliminate the interference under large and medium loads. As a derivative control algorithm of speed synchronization, this idea is applied to the multichannel loading system [33]. The difference is that as the number of loading channels increases, the influence of coupling force becomes more and more prominent, severely reducing the loading accuracy, especially when increasing the loading frequency. This also puts higher demands on the decoupling control method.

In this paper, a new less sensors motion synchronous composite control method is proposed. On the basis of using the valve control signals and force commands to ensure passive motion coordination, the dynamic tracking characteristics of multi-loading system are improved. It can be found from the comparison of the experiment results that the control scheme can effectively eliminate the coupling disturbance of each channel and has a good compensation control effect.

In the three-channels loading test, the negative coupling force of Channel 3 is largest. The phase lag of Channel 3 is very severe because Channel 3 is affected by the forces of all channels. After the compensation of valve control signals and force command signals, the error caused by the coupling force is significantly reduced.

## 7. Conclusions

From above statement, the following conclusions can be obtained within the decoupling strategy on multichannel loading system.

1. The novel motion synchronous composite decoupling control method can realize decoupling in the case of linear loading with fewer sensors. Yet the traditional decoupling control method requires the addition of displacement, velocity, and acceleration sensors or high-order differentiation, which will increase the complexity of measurement system.
2. The composite control method uses only the command signal and control signal to achieve multichannel decoupling under the condition of increasing the loading frequency. It is able to improve the dynamic tracking accuracy of the multichannel loading system. It is of great significance that this decouple method could guarantee control accuracy in the case where the test frequency is increased. The method is able to maintain accurate loading of the force at increasing frequency, so that the loading time can be shortened.

## Figures and Tables

**Table 1:** Symbol definitions and main parameters.

Symbol	Definition	Value and Unit
$Q_{L}$	total flow from servo valve	/
$K_{q}$	flow gain of servo valve	3 $m^{2}$
$x_{v}$	servo valve spool displacement	/
$K_{c}$	flow pressure coefficient of the servo valve	/
$p_{f}$	load pressure of system	/
A	effective area of the piston	14.6$\;\times \;10^{-4}{\;m}^{2}$
$x_{d}$	displacements of the piston rod	/
V	total volume of the hydraulic cylinder	4$\;\times \;10^{-6}{\;m}^{3}$
E	elastic modulus	7$\;\times \;10^{8}\;\mathrm{pa}$
$C_{s}$	comprehensive leakage coefficient	4.7$\;\times \;10^{-13}{\;m}^{5}/\left(N\cdot s\right)$
$M_{a}$	mass of the piston rod	/
$B_{a}$	viscous damping coefficient	/
$K_{a}$	stiffness coefficient of the force sensors	/
$x_{d}^{\prime }$	displacement of the sensor’s lower surface	/
$M_{L}$	the mass of the concentrated mass of load	10 kg
$B_{L}$	damping coefficient load	1 N·s/m
$K_{L}$	stiffness coefficient of load	$10^{6}\;N/m$
